# Labelling indices in human tumours: to apply corrections or not – that is the question

**DOI:** 10.1038/sj.bjc.6690574

**Published:** 1999-07

**Authors:** C Bergström, A Begg, R Palmqvist, A Waites, J Denekamp

**Affiliations:** Department of Oncology and Pathology, Umeå University, SE-901 85 Umeå, Sweden; Department of Experimental Therapy, Netherlands Cancer Institute, Amsterdam, The Netherlands

**Keywords:** proliferation, human tumours, labelling index, colorectal cancers, IUDR

## Abstract

The advent of halogenated pyrimidines (bromodeoxyuridine, BrdU; idoxuridine, IdU) and antibodies to recognize them has opened new horizons for the measurement of proliferation in human tumours. These precursors of DNA can be given to patients and a single biopsy can be taken to measure in a flow cytometer both the fraction of labelled cells and their rate of movement through the S phase. From these two parameters the potential doubling time, T_POT_, can be calculated. To measure both parameters simultaneously a compromise is made in the time of assessing the labelling index (LI). LI should ideally be assessed after a very short interval, e.g. 0.5–1 h, to avoid the contaminating influence of any cells dividing between injection and biopsy. However, an interval of 4–8 h is considered necessary to assess T_S_ from the relative movement of cells through the S phase. Several techniques exist to correct for cell division if the interval is long. The simplest correction, which only corrects for the division of labelled cells, is most widely used. Downward correction factors of at least 10% are commonly applied, reducing the observed LI values. In this paper we illustrate graphically the dependence of the appropriate correction factor on various cell kinetic parameters. The duration of G2 is the most critical parameter for both the size and direction of any correction factor. The G2 phase has previously been shown to be about three times longer in human tumours than in rodents. If G2+M is as long as 6 h, the main artefact of the intervals between injection and biopsy up to 7 h is that the observed LI is too low because of division of unlabelled G2 cells. A correction of up to 10% is needed but in an upward direction. A nomogram of probable correction factors as a function of sampling interval is provided. We show from flow cytometric data that G2+M may be shorter than 4 h for head and neck tumours. It is recommended that the correction factor established by gating the flow histogram should always be checked against this nomogram, or that no correction factor should be applied. We have used this mathematical approach to re-evaluate two sets of published LI data for rectal and colorectal tumours. We show that the mathematical correction of each data point leads to a 30% increase in the median value, compared to the simple gating procedure. We question whether other of the published series of LI values gained with BrdU or IdU may also substantially underestimate the true LI values, if a simple gating procedure has been used in an attempt to reduce the impact of divided S phase cells. © 1999 Cancer Research Campaign


*British Journal of Cancer* (1999) **80**(10), 1635–1643
© 1999 Cancer Research Campaign
Article no. bjoc.1999.0574

### Labelling indices in human tumours: to apply corrections or not – that is the question

**C Bergström[1], A Begg[3], R Palmqvist[2], A Waites[1] and J Denekamp[1]**

Department of [1]Oncology and [2]Pathology, Umeå University, SE-901 85 Umeå, Sweden; and [3]Department of Experimental Therapy, Netherlands Cancer Institute, Amsterdam, The Netherlands

**Summary** The advent of halogenated pyrimidines (bromodeoxyuridine, BrdU; idoxuridine, IdU) and antibodies to recognize them has opened new horizons for the measurement of proliferation in human tumours. These precursors of DNA can be given to patients and a single biopsy can be taken to measure in a flow cytometer both the fraction of labelled cells and their rate of movement through the S phase. From these two parameters the potential doubling time, $T_{POT}$, can be calculated. To measure both parameters simultaneously a compromise is made in the time of assessing the labelling index (LI). LI should ideally be assessed after a very short interval, e.g. 0.5–1 h, to avoid the contaminating influence of any cells dividing between injection and biopsy. However, an interval of 4–8 h is considered necessary to assess $T_S$ from the relative movement of cells through the S phase. Several techniques exist to correct for cell division if the interval is long. The simplest correction, which only corrects for the division of labelled cells, is most widely used. Downward correction factors of at least 10% are commonly applied, reducing the observed LI values. In this paper we illustrate graphically the dependence of the appropriate correction factor on various cell kinetic parameters. The duration of G2 is the most critical parameter for both the size and direction of any correction factor. The G2 phase has previously been shown to be about three times longer in human tumours than in rodents. If G2+M is as long as 6 h, the main artefact of the intervals between injection and biopsy up to 7 h is that the observed LI is too low because of division of unlabelled G2 cells. A correction of up to 10% is needed but in an upward direction. A nomogram of probable correction factors as a function of sampling interval is provided. We show from flow cytometric data that G2+M may be shorter than 4 h for head and neck tumours. It is recommended that the correction factor established by gating the flow histogram should always be checked against this nomogram, or that no correction factor should be applied. We have used this mathematical approach to re-evaluate two sets of published LI data for rectal and colorectal tumours. We show that the mathematical correction of each data point leads to a 30% increase in the median value, compared to the simple gating procedure. We question whether other of the published series of LI values gained with BrdU or IdU may also substantially underestimate the true LI values, if a simple gating procedure has been used in an attempt to reduce the impact of divided S phase cells.

**Keywords:** proliferation; human tumours; labelling index; colorectal cancers; IUDR

Techniques to measure the proliferation rate of human tumours in situ have been sought for decades. Historically, estimates were based on the fraction of mitoses visible in histological specimens. In the 1950s, radioactive precursors of DNA were used to identify the cells preparing for division and the four separate phases of the cell cycle were identified, G1, S, G2 and M (Howard and Pelc, 1953). This was followed in the 1960s by techniques to measure the cell cycle, the growth fraction and the rate of cell loss in order to provide a complete description of the growth characteristics of experimental and human tumours (reviewed in Steel, 1977). Unfortunately, these techniques required multiple biopsies and the DNA targeted radio-isotopes were not suitable for widespread use in humans, because of their genotoxic potential.

In the 1970s, flow cytometers were manufactured and the fraction of cells in the S phase could be identified without administering precursors, by staining cells for their DNA content and counting those between diploid and tetraploid DNA content. However, this does not give kinetic information about transit times and cannot distinguish between active and quiescent S phase cells. Eventually, in the early 1980s, non-radioactive halogenated precursors were developed that could, when bound into DNA, be identified using monoclonal antibodies with fluorescent tags (Gratzner et al, 1982; Dolbeare and Gray, 1983). It was realized that this provided the potential to measure both the fraction of cells actively synthesizing DNA and their rate of progress through the S phase from a single biopsy sample (Begg et al, 1985). It was called the relative movement assay. This made it possible, at last, to obtain from a single biopsy in patients the two independent parameters, Ts and labelling index (LI), that are needed to estimate the potential doubling time, $T_{pot}$, of a tumour.

$$Tpot = \lambda \cdot \frac{Ts}{LI} \qquad \text{(Steel, 1977)} \qquad (1)$$

λ is a correction factor for the non-linear age distribution, and is often arbitrarily (as in our centre) set at 0.8. There are, inevitably, a number of assumptions in applying this technique, and a certain degree of compromise in the time interval that must be used between labelling and sampling the cells. A very short interval (0.5–1.0 h) is optimal for determining LI, but an interval that is

*Received 25 September 1998*
*Revised 22 January 1999*
*Accepted 4 February 1999*

*Correspondence to:* C Bergström about half $T_S$ is optimal for defining the relative movement of labelled S phase cells towards a G2 content of DNA. An interval of 4–8 h is generally recommended. For cells in vitro or in rodent tumours, this interval is much longer than the phases (G2 plus M). This interval would, in such systems, allow labelled cells to transit these phases and divide, thus adding extra labelled cells to the population. This potential artefact in the assessment of LI was recognized early on, and several methods have been offered to solve it. The first was a simple and practical gating procedure of the bivariate histogram (Begg et al, 1988). The labelled cells are assessed in the flow cytometry histogram, and a decision is made about which cells have transited G2 and mitosis and re-entered G1 as two cells. This group of cells is then gated and counted separately, divided by two and subtracted from both the labelled cells and the total cells to obtain a corrected LI (see Begg, 1989, for details). No account was taken of the division of unlabelled cells leaving G2 and doubling as they go through mitosis, because for cells in vitro and for animal tumours these phases are very short.

A modification of this formula was proposed by Brons et al (1992) to take into consideration the division of unlabelled G2 cells within an interval corresponding to G2+M. Although this is more accurate, the simple formula is more commonly applied. It has been pointed out that the simple (Begg) correction is inappropriate for very short sampling times or for histograms where there is not clear evidence of two separate sub-populations in the bivariate histograms (Wilson et al, 1988; Begg, 1989). However, in practice, in many centres as in ours it may be applied somewhat too frequently.

An alternative and more sophisticated approach is to use a much more complex mathematical treatment of the measured LI. This involves assumptions or prior knowledge about the phase duration and the form of the growth curves (White et al, 1990; Johansson et al, 1998). The mathematical correction is more accurate but appears somewhat complex and has not been widely adopted by flow cytometry operators.

In this paper we demonstrate graphically the correction factors that should theoretically be applied for various assumptions about phase durations. We show the consequences for different values of LI. We have used the same mathematical model as that of White et al (1990). We have then used this approach to re-evaluate two sets of our own published proliferation data for colorectal cancers. We demonstrate the difference if the commonly used simple correction, or the mathematical formula based on best estimates of the duration of G2 and S in human tumours is applied.

### MATERIALS AND METHODS

There are two parts to this study: a mathematical component, and a practical analysis of patient data.

### Mathematical modelling

A flash label is assumed to be administered which marks only those cells actively synthesizing DNA. As the cells progress around the cell cycle, first unlabelled cells (originally in G2 and M phase) then labelled cells (originally in S phase) divide, and therefore the fraction of labelled cells ($f^l$) varies with time. As described by White et al (1990), this fraction can be calculated at any time $t$ after labelling, according to

$$f^l(t) = \begin{cases} e^{c(T_{G_2+M}-t)}\,(e^{cT_S}-1) & \text{if } t \le T_{G_2+M} \\ 1 + e^{c(T_{G_2+M}-t)}\,(e^{cT_S}-2) & \text{if } T_{G_2+M} \le t \le T_S + T_{G_2+\mathrm{M}} \\ 2e^{c(T_{G_2+M}-t)}\,(e^{cT_S}-1) & \text{if } T_S + T_{G_2+M} \le t \le T_C + T_{G_2+M} \end{cases} \tag{2}$$

where $T_C$, $T_{G2+M}$ and $T_S$ are the durations of the total cell cycle, or the individual phases (G2+M) and S, which must be estimated experimentally or assumed. If $p$ is the probability that a new cell is proliferating, then

$$c = \frac{\ln(2p)}{T_C}\,. \tag{3}$$

$p = 1$ corresponds to a growth fraction of 1, which is what we have assumed in this study. If a growth fraction less than unity is assumed it does not change any of the conclusions for time intervals less than G2+M+S. Equation (2) can then be used to calculate $f^l(t)$ for any time $t$ after injecting the stain. It is also possible to solve for $T_C$ for a known $f^l(t)$ and $t$, with an assumed $T_S$ and $T_{G_2+M}$. Then, by setting $t = 0$, one can calculate the true LI.

### Analysis of human tumours

We have taken two sets of flow cytometry data for human tumours that have already been published (Bergström et al, 1998; Palmqvist et al, 1998). We have re-considered the data from bivariate histograms, i.e. the corrected LI and the correction factor. These were originally obtained by gating labelled cells that were considered by the FCM operator to have divided in the interval between administering the precursor and surgical excision. Using the simple practical correction, these gated labelled G1 cells were halved (to correct for division) and subtracted from both the numerator and the denominator to obtain LI corrected. The two data sets have now been re-analysed. Using the actual raw count of LI a theoretical correction was applied from equation 2, calculated for the specific time from injection to surgery for that sample.

In order to undertake this more complex mathematical modelling it is necessary to specify the appropriate values for the cell cycle phase durations. These are listed in Table 1 for spontaneous human tumours and for a range of experimental models, determined by the per cent labelled mitosis method (Steel, 1977). The combined duration of the phases (G2 and mitosis) is the most important parameter for the present purposes, since cells labelled while in the S phase must transit these phases before appearing as two cells in G1. Using the per cent labelled mitosis curve method, $T_{G2+M}$ was found to be approximately three times longer in human

**Table 1** Average estimates of cell cycle phase durations obtained with the per cent labelled mitosis technique in solid tumours (from Steel, 1977)

| Tumour type | G1 (h) | S (h) | G2[a] (h) | LI (%) |
|---|---|---|---|---|
| Human tumours | 22 (8–38) | 16 (10–24) | 6 (2–10) | 19 (4–29) |
| Frequently passaged mouse tumours | 4 (2–7) | 9 (6–12) | 2 (1–5) | 32 (12–68) |
| Frequently passaged tumours in rats and hamsters | 11 (4–39) | 8 (5–10) | 3 (1–4) | 20 (10–36) |
| Early transplants of tumours in rats and mice | 13 (4–48) | 11 (4–18) | 2 (1–4) | 18 (5–45) |
| Primary tumours (animals) | 13 (2–36) | 8 (5–11) | 3 (1–6) | 14 (6–31) |

[a]Time in mitosis is approximately 1 h in experimental systems. Arithmetic mean of quoted values with range shown in brackets.

*© 1999 Cancer Research Campaign*

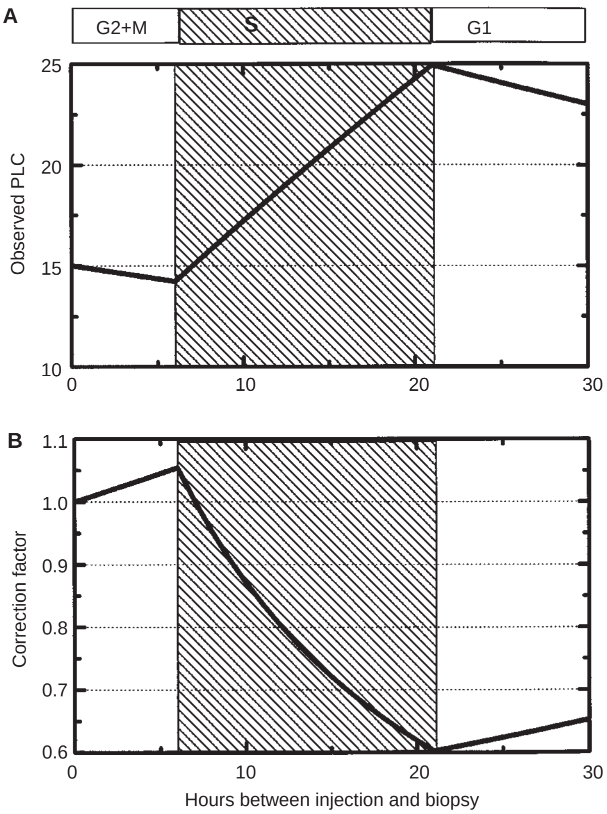

**Figure 1** Schematic illustration of the influence of cell division in the interval between injection of the labelled DNA precursor and surgery. (**A**) The fluctuation in the observed LI with time for an assumed initial $LI_0$ of 15%, if $T_{G2+M}$ = 6 h and $T_S$ = 15 h. Initially, the observed LI drops, as unlabelled G2 cells divide, and then increases as cells originally labelled in S pass through mitosis. (**B**) This leads to fluctuations in the factor needed to convert the observed LI at time T to the true LI at time = 0. An upward correction (> 1.0) is needed at times shorter than $T_{G2+M}$.

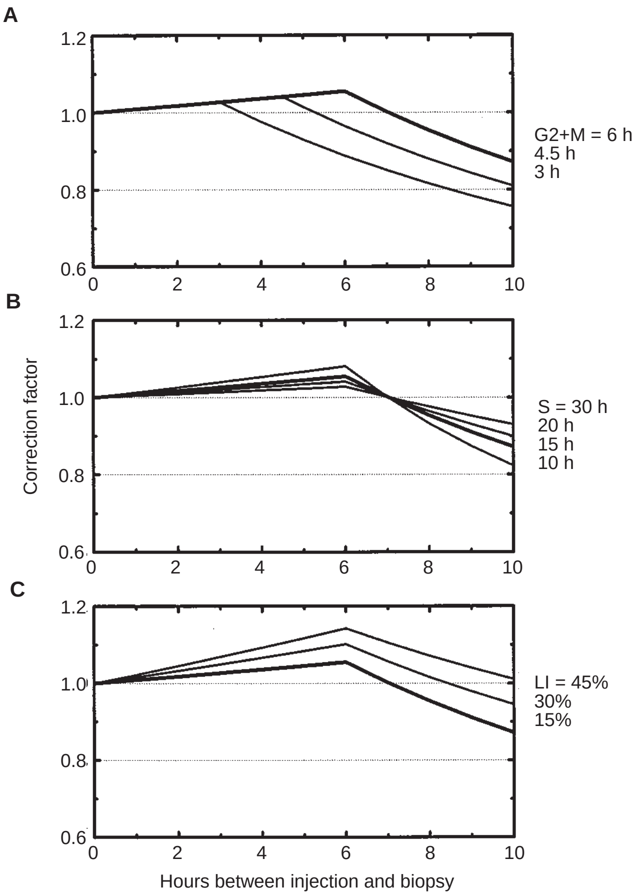

**Figure 2** Schematic illustration of the influence on the correction factor for different time intervals of various cell kinetic parameters. (**A**) Variation of $T_{G2+M}$ for a constant $T_S$ = 15 h, $LI_0$ = 15% influences both the timing of the inversion from a positive to a negative correction factor, and the maximum magnitude of the positive correction factor. (**B**) Variation of $T_S$ for a constant $T_{G2+M}$ = 6 h, $LI_0$ = 15% has a direct influence on the magnitude of the correction factor, but not on the timing of the inversion. (**C**) Variation of initial LI for constant $T_S$ = 15 h, $T_{G2+M}$ = 6 h. The magnitude of the upward correction to counteract addition of unlabelled cells by division is increased with higher values of LI

tumours (mean value 6 h) than in rodents. Using flow cytometry techniques somewhat shorter $T_{G2+M}$ estimates have been found, e.g. 4.5 h (Begg, 1989). A range of possible $T_{G2+M}$ values have therefore been used for our calculations.

### RESULTS

Figure 1 illustrates the cyclic fluctuations in the observed LI values if the interval between labelling and sampling is varied between 1 h and 30 h. In this first example we have used representative values for LI of 15%, $T_{G2+M}$ of 6 h and $T_S$ of 15 h. The upper panel (Figure 1A) shows that the LI falls from the starting value of 15% for a period corresponding to $T_{G2+M}$, during which time unlabelled cells are being added by cell division. After 6 h this reverses and the LI then increases over the next 15 h as labelled cells divide. Subsequently, as G1 cells begin to enter mitosis, the unlabelled cells increase again. The observed LI falls back towards the starting value, which it will reach after one complete cell cycle or potential doubling time unless there is a differential cell loss from a specific phase. The lower panel shows the correction factor that needs to be applied to convert the observed value at any particular time back to the original starting value (of 15%). It is the converse of the LI fluctuations. Figure 1B illustrates clearly that a factor above unity is needed to correct the LI value upward over the first 6 h (i.e. $T_{G2+M}$) because of addition by division of unlabelled cells. In this example, only after 7 h is a downward correction needed.

Figure 2 shows how the correction factor depends upon the chosen parameters. In general, the default values for these calculations have been set at $LI_0$ = 15%, $T_{G2+M}$ = 6 h and $T_S$ = 15 h. In each panel two of these parameters are kept constant and the third is varied systematically to determine its impact. The graphical display is now limited to the region of clinical interest, i.e. the first 10 h. (The recommended interval between labelling and obtaining the tumour specimen by biopsy or surgery is 4–8 h.)

Figure 2A shows the major influence of the duration of G2+M. Unlabelled cells transit mitosis and produce an artificial reduction in the observed LI for a period that is a little longer than $T_{G2+M}$. Thus a positive correction is needed to increase $LI_T$ to its original value for longer intervals if the duration of $T_{G2+M}$ is longer. The correction factor is small, and does not reach 10% in these examples.

Figure 2B shows that the value of $T_S$ has no influence on the time period for which an upward correction is needed, but it does have an influence on the magnitude of the actual correction factor.

*© 1999 Cancer Research Campaign*

**Table 2** Nomogram to illustrate correction factors calculated for a range of LI values and sampling times

| $T_{G2+M}$ = 6 h<br>LI = | 5% | 10% | 15% | 20% | 25% | 30% | 35% |
|---|---|---|---|---|---|---|---|
| Time | | | | | | | |
| 2 h | 1.01 | 1.02 | 1.02 | 1.03 | 1.03 | 1.04 | 1.04 |
| 3 h | 1.01 | 1.03 | 1.03 | 1.04 | 1.04 | 1.05 | 1.06 |
| 4 h | 1.03 | 1.03 | 1.04 | 1.05 | 1.06 | 1.07 | 1.08 |
| 5 h | 1.03 | 1.04 | 1.05 | 1.06 | 1.08 | 1.09 | 1.10 |
| 6 h | 1.04 | 1.04 | 1.06 | 1.08 | 1.10 | 1.11 | 1.13 |
| 7 h | 0.98 | 0.99 | 1.01 | 1.02 | 1.05 | 1.06 | 1.08 |
| 8 h | 0.92 | 0.93 | 0.96 | 0.97 | 1.00 | 1.02 | 1.04 |
| $T_{G2+M}$ = 4.5 h<br>LI = | 5% | 10% | 15% | 20% | 25% | 30% | 35% |
| Time | | | | | | | |
| 2 h | 1.01 | 1.02 | 1.02 | 1.03 | 1.03 | 1.04 | 1.04 |
| 3 h | 1.03 | 1.03 | 1.03 | 1.04 | 1.05 | 1.05 | 1.06 |
| 4 h | 1.03 | 1.03 | 1.04 | 1.05 | 1.06 | 1.07 | 1.08 |
| 5 h | 1.00 | 1.00 | 1.02 | 1.03 | 1.05 | 1.06 | 1.07 |
| 6 h | 0.94 | 0.95 | 0.97 | 0.98 | 1.00 | 1.02 | 1.03 |
| 7 h | 0.89 | 0.90 | 0.92 | 0.94 | 0.96 | 0.97 | 0.99 |
| 8 h | 0.85 | 0.86 | 0.87 | 0.89 | 0.92 | 0.93 | 0.95 |
| $T_{G2+M}$ = 3 h<br>LI = | 5% | 10% | 15% | 20% | 25% | 30% | 35% |
| Time | | | | | | | |
| 2 h | 1.02 | 1.02 | 1.03 | 1.03 | 1.03 | 1.04 | 1.04 |
| 3 h | 1.03 | 1.02 | 1.03 | 1.04 | 1.05 | 1.05 | 1.06 |
| 4 h | 0.97 | 0.97 | 0.98 | 0.99 | 1.00 | 1.01 | 1.02 |
| 5 h | 0.91 | 0.92 | 0.93 | 0.94 | 0.96 | 0.97 | 0.98 |
| 6 h | 0.87 | 0.87 | 0.88 | 0.90 | 0.92 | 0.93 | 0.95 |
| 7 h | 0.82 | 0.83 | 0.84 | 0.86 | 0.88 | 0.90 | 0.91 |
| 8 h | 0.77 | 0.79 | 0.80 | 0.82 | 0.84 | 0.86 | 0.88 |

For this threefold change in $T_S$ the correction factors are all small (≤ 10%), but positive, for a little longer than the duration of G2+M.

Figure 2C shows the impact of the choice of $LI_0$ for these schematic illustrations. The correction factor in the early time intervals varies in proportion to the fraction of unlabelled cells at the start. For higher $LI_0$ values, unlabelled cells are rarer, and the addition of extra unlabelled cells from G2 has a greater impact. The magnitude of the correction is directly proportional to $LI_0$ and stays positive for a longer time with higher labelling indices.

Table 2 is a nomogram which shows the correction factors that should be applied for intervals of 2–8 h for initial LI ranging from 5–35%. This has been constructed on the assumption of phase durations being $T_S$ = 15 h and $T_{G2+M}$ = 6 h in the upper panel, 4.5 h in the middle panel and 3.0 h in the lower panel. The important feature of Table 2 is that almost all the correction factors listed are greater than unity and illustrate the frequent need to correct upwards, not downwards, especially if $T_{G2+M}$ is longer than 3 h.

Figure 3 illustrates two sets of experimental data obtained with a flow cytometer from individual patients in Umeå after surgical excision of colon tumours. In the left-hand panels the tumour was removed at a very short interval (1.2 h), whereas in the right-hand panels the sample was taken 6.4 h (i.e. within the recommended range) after administering 100 mg IdU intravenously to the patient. The vertical axis reflects fluorescence which is proportional to IdU incorporation. The horizontal axis represents propidium iodide fluorescence, which indicates the DNA content. The frames that are shown were those applied routinely by the flow cytometry operator, who had no knowledge of the interval between injection and surgery. Since it has been specified that the appearance of a subset of divided labelled cells is a necessary 'quality control' feature for the relative movement assay (Begg, 1989) an effort is always made to identify and gate out the G1 cells in the bivariate histograms. In the left-hand histogram, although there is no clear margin between the two gates, they have nevertheless been defined, setting a gate over the G1 peak. We now believe that the frame applied in the left hand panel is inappropriate because it assumes cell division of labelled cells in an interval that is too short to allow that. These cells must be undivided early S phase labelled cells. In this very short interval between labelling and surgical excision, it is extremely unlikely that any labelled cells could have traversed G2 and mitosis. In the right-hand panel, by contrast, there is a clear zone between the two clouds of labelled cells, and this histogram shows that a significant fraction of S phase cells have divided in this tumour within 6.4 h. These gating procedures, as shown, are routinely applied within our pathology department.

We have taken two recently published sets of data from our institution in which we have re-evaluated each flow cytometry histogram to obtain each raw uncorrected value of LI. We have then replaced the simple gating correction factors with those calculated as in Table 2. Figure 4 shows the factors that were originally applied by FCM gating to correct each data point in these two published series of colon and rectal tumours. They range from 0.63 to 0.95 and show remarkably little dependence upon the time interval between injection and tumour excision. The bold solid line shows the correction factor that should theoretically apply if $T_{G2+M}$ = 6 h, $T_S$ = 15 h and $LI_0$ = 15%. The other lines show the correction factors if $T_{G2+M}$ is as short as 4.5 or even 3 h. It is clear that almost all of the correction factors that were applied by the simple gating procedure are in disagreement with the more precise mathematical prediction. Even if the G2+M were as short as 3 h (lower dashed line), all but three of the correction factors should not be as low as those that have in practice been applied.

Figure 5 illustrates the individual LI values obtained with the two different methods of correcting for cell division. In the left hand panels the data are illustrated, divided according to the time interval, for short, average and long intervals. There is a consistent deviation of the points away from the 1:1 correlation independent of the interval between injection and surgery. The right hand panels show that the rank order is similar but not identical for the LI corrected by the two methods.

Figure 6 illustrates the consequence of these two different approaches to correcting LI and compares them with the raw data. Cumulative frequencies of LI are shown, and the median value is indicated at 50% on each curve. Figure 6A represents 34 rectal carcinomas and Figure 6B 53 colon carcinomas. The median LI values corrected by the mathematical modelling technique are almost identical to the raw data. However, the median values after correction by the FCM gating technique are 27–31% lower compared with the original raw data. The progressive separation of the curves at higher values of LI shows the increasing influence of the addition of unlabelled cells by division at early times if they are relatively rare in the population. Figure 6 clearly demonstrates that for these two sets of data the raw uncorrected LI are more accurate than those to which the simple FCM gating correction has been applied.

The means and ranges derived from the publications where these data were originally reported are summarized in Table 3 (rectal tumours, Bergström et al, 1998, and colorectal tumours,

 *© 1999 Cancer Research Campaign*

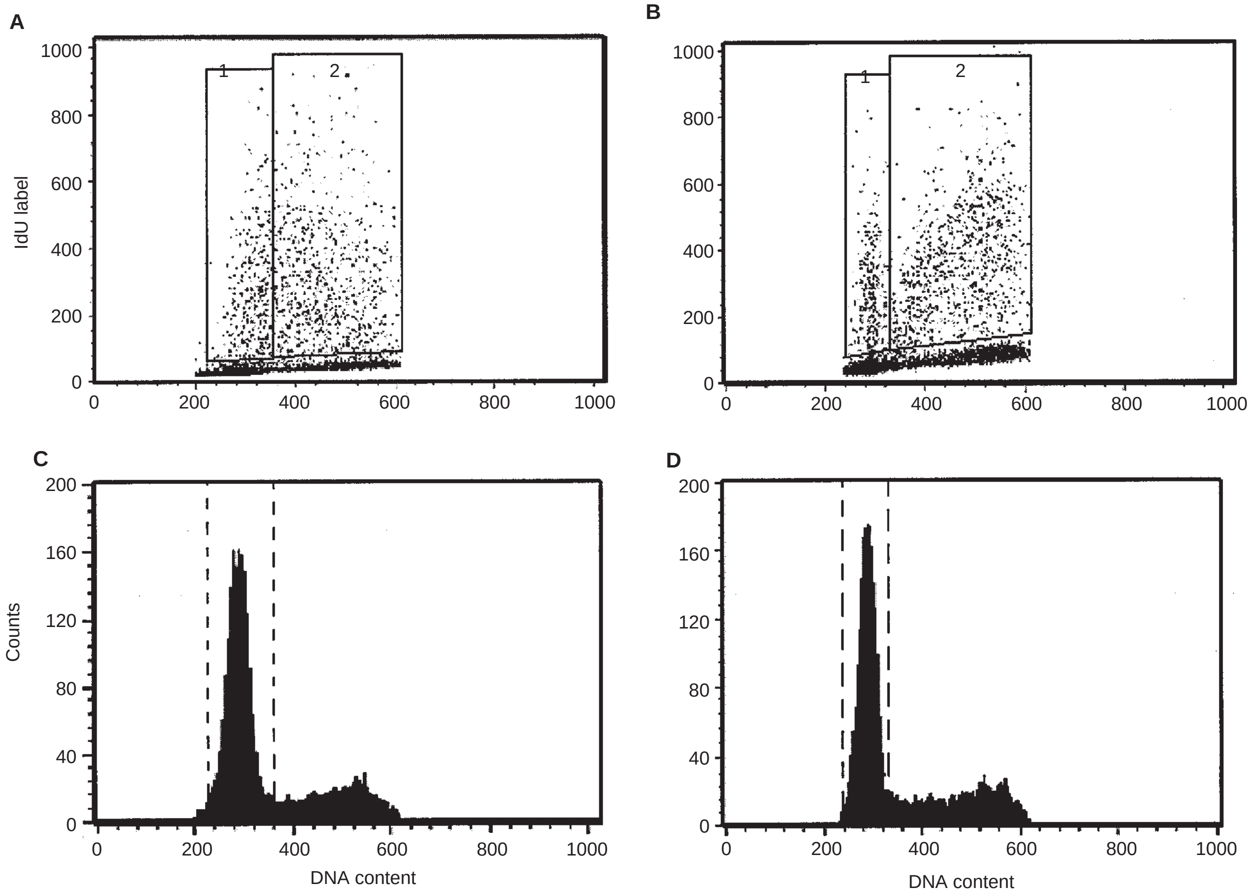

**Figure 3** Two examples of bivariate flow cytometry histograms of colon carcinomas from the series of colorectal cancers published by Palmqvist et al (1998). Corresponding DNA histograms are shown below. The gates applied according to the routine procedures are indicated. (**A**, **C**) short interval of 1.2 h. (**B**, **D**) long interval of 6.4 h. A subset of idoxuridine (IdU) labelled cells was gated by the operator in both panels as representing divided labelled cells (gate 1), and correction factors of 0.89 were derived from both histograms.

**Table 3** Summary of labelling indices from two published studies, together with the FCM gating correction and three theoretically calculated values (using equation 2)

| | 53 colon cancer patients (from Palmqvist et al, 1998) | | | 34 rectal cancer patients (from Palmqvist et al, 1998; Bergström et al, 1998) | | |
|---|---|---|---|---|---|---|
| | **Mean** | **Median** | **Range** | **Mean** | **Median** | **Range** |
| Observed plc (raw data) | 13.3 | 11.6 | 2.4–42.6 | 16.7 | 15.1 | 2.6–55.4 |
| Mathematical correction if $T_{G2+M} = 3$ | 12.4 | 9.8 | 2.1–40.3 | 15.8 | 13.4 | 2.1–56.3 |
| $T_{G2+M} = 4.5$ | 13.3 | 10.6 | 2.3–40.3 | 16.9 | 14.7 | 2.4–61.1 |
| $T_{G2+M} = 6$ | 13.8 | 11.5 | 2.5–48.0 | 17.7 | 15.6 | 2.7–65.6 |
| With FCM gated CF | 9.7 | 8.5 | 1.8–32.8 | 12.3 | 10.8 | 1.7–45.7 |

Palmqvist et al, 1998). The choice of correction factor makes a substantial difference to the conclusion about the mean LI and the range in both these data sets. The mathematically corrected LI values do not change much even if G2+M is varied between 3 and 6 h. They are all quite close to the uncorrected value and differ markedly from those derived with a simple FCM correction. This indicates that less error is introduced if no correction factor is applied.

### DISCUSSION

The technical innovation of being able to use a single biopsy to simultaneously measure Ts and LI has made it possible to accumulate a large amount of data on human tumour cell kinetics. Many thousands of patients have now received one of the halogenated pyrimidines and most of the studies have shown that the LI is very variable from patient to patient, and from one histological type and

*© 1999 Cancer Research Campaign*

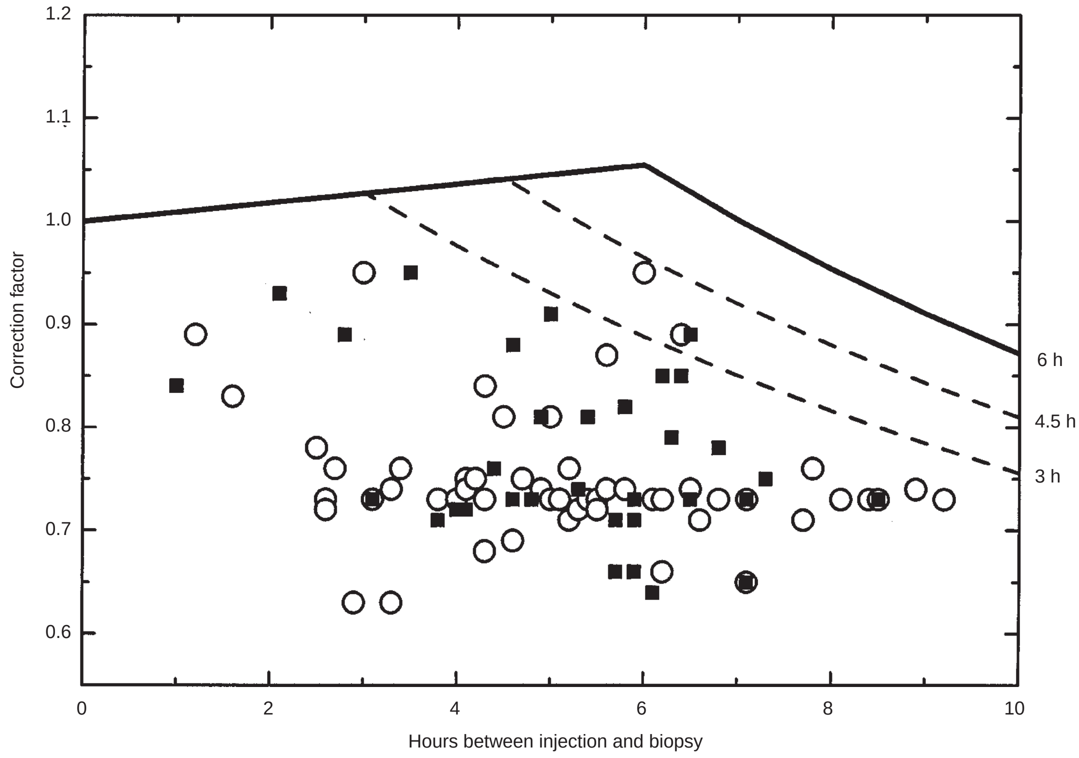

**Figure 4** Comparison of the correction factors derived from the simplified gating of the individual flow cytometry histograms (data points) with those predicted from the theoretical analysis. Three curves are shown for $T_{G2+M}$ durations of 3, 4.5 and 6 h. Most of the FCM derived corrections are far below the theoretically derived values and show no significant trend with time site to another (e.g. Rew et al, 1991; Bennett et al, 1992; Wilson et al, 1993*a*, 1993*b*; Begg, 1995; Terry et al, 1995; Bergström et al, 1998; Palmqvist et al, 1998). The flow cytometric estimates of the potential doubling time from these relative movement assays show that the median $T_{pot}$ is around 4–7 days for most tumour types, but with a spread from 1 to 30 or more days. The duration of $T_{pot}$ is directly linked to the estimate of LI. Thus any underestimate of LI, because of the inappropriate use of the simple gating correction factor would translate into a corresponding overestimate of the potential doubling time. This is a minor variation compared with the 10- to 20-fold difference from volume doubling times but still may be important for practical purposes. The absolute values of $T_{pot}$ are now being built into many predictive models of the consequences of fractionation using shorter treatment schedules (e.g. Fowler and Lindström, 1992). Tumours are sometimes classified as 'fast' if their $T_{pot}$ value is shorter than 5 days and 'slow' if it is longer. The systematic 'down correction' of LI would change the proportions in these two categories and hence the need for selection of patients for accelerated regimes.

Several large studies are in progress to evaluate the relationship between the estimate of LI or $T_{pot}$ and the outcome of treatment with either a conventional or an accelerated regime (e.g. Begg et al, 1999; P Coucke et al, unpublished data). These are designed to determine whether these kinetic parameters are useful prognostic or predictive markers. The ultimate goal is to be able to identify those patients at most risk of proliferation during a course of therapy and select those for an accelerated regime. For this reason it is very important to avoid random or systematic errors creeping into the measurements, or differences in analytical procedures from one centre to another.

It has long been recognized that the long interval needed between injection and sampling of the tumour for the relative movement assay may necessitate a correction of LI (Begg, 1989). The simple practical solution that is commonly applied, however, totally ignores the contribution of unlabelled G2 cells as they divide. It focuses only on the artefact of additional labelled cells, as those from the labelled compartment transit through G2 and mitosis. It has been stressed that this is inappropriate if the time between injection and sampling is too short and will give an underestimate (Begg, 1988; Wilson et al, 1988). It then becomes very important to consider how short is too short, and for this Figure 2 shows that the duration of G2+M is the crucial parameter.

Table 1, containing data from the comprehensive review by Steel (1977), shows that the estimates obtained from human tumours in the 1970s, using the very detailed studies of multiple biopsies after administering tritiated thymidine, range from 2 to 10 h with an average of 6 h. Begg (1989), however, deduced a value slightly lower than this average. He considered the fraction of tumours with clear movement of labelled cells into G1 as a function of the time after administering bromodeoxyuridine (BrdU), and reported an average value of 4.5 h.

Figure 7 summarizes three sets of data from which the duration of G2+M can also be derived. One set is from a single centre (Amsterdam) assessment of all the tumours entered into the multicentre randomized EORTC accelerated radiotherapy trial (Begg et al, 1998). It shows that about 80% of the tumours are considered to

 © 1999 Cancer Research Campaign

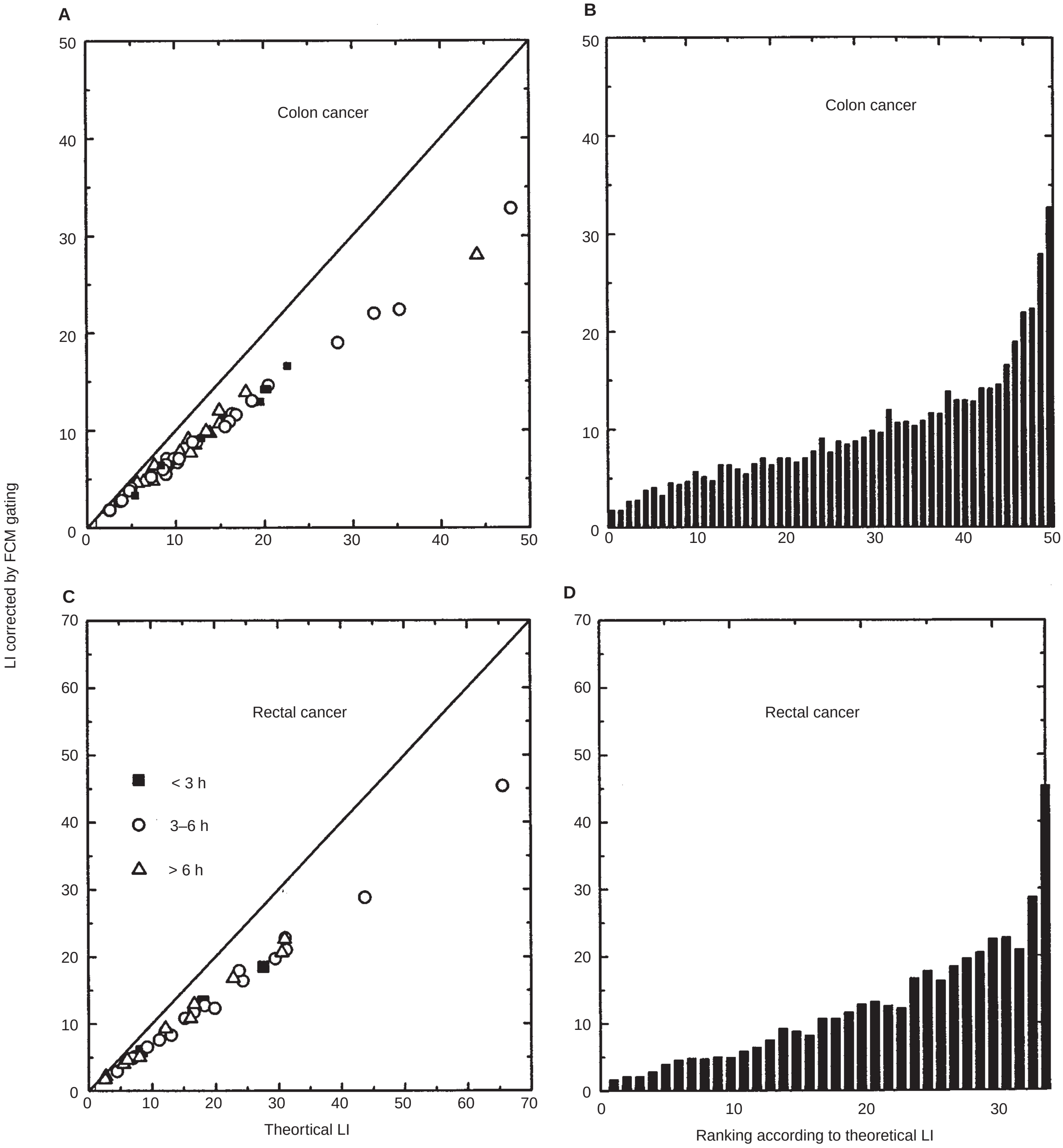

**Figure 5** Individual LI values of 34 rectal tumours and 53 colon tumours obtained with the two different methods of correcting for cell division. (**A**, **C**) The time interval between label and biopsy for individual tumours is shown (■: < 3 h, ○: 3–6 h, ▲: > 6 h). (**B**, **D**) The size of the bars indicates FCM corrected LI. The bars are ranked according to theoretically calculated LI

have divided labelled cells in G1 by 4 h and 100% by 4.5 h. The second set of data comes from a single-centre study in Cairo by Awwad and colleagues, for which all the data have been analysed in Amsterdam (unpublished). It shows 85% of the tumours having been described as having labelled divided cells at 4 and 5 h, and all by 5.5 h. Both data sets would imply a G2+M that is shorter than 4 h in many human tumours. The Umeå data in Figure 6 are clearly in disagreement with those from Amsterdam since they show that all tumours were considered to have labelled G1 cells regardless of the interval between labelling and sampling. This is biologically unrealistic.

The practice of gating divided labelled cells seems to differ from centre to centre. The details of the method and the resultant correction factor is not quoted in publications, and is therefore

*© 1999 Cancer Research Campaign*

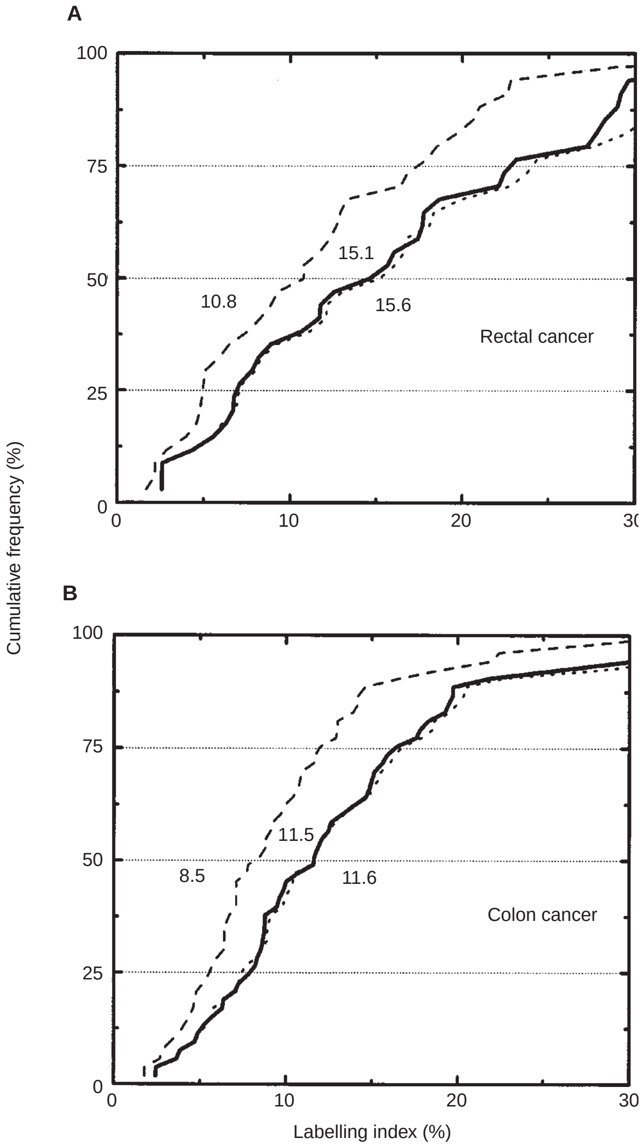

**Figure 6** Cumulative frequency plots of the LI for 28 rectal tumours and 53 colon tumours if expressed as the raw data from flow cytometry (—), with a theoretical correction applied to each value (assuming $T_{G2+M}$ = 6 h, $T_S$ = 15 h) (. . .), or an adjustment by gating for divided labelled cells (- - -). The raw data are much closer to the mathematically corrected $LI_0$ values than those adjusted via the flow histogram. The simple correction leads to a considerable underestimate of LI, especially at the higher values

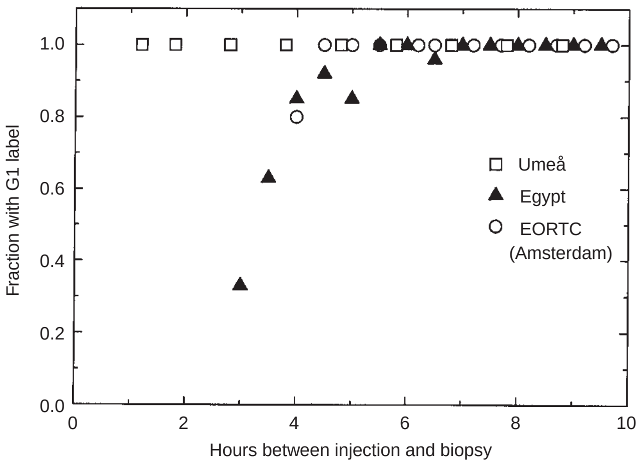

**Figure 7** Percentage of cells with divided labelled cells in each time interval as a function of the time of sampling. Three series are shown: ○: head and neck cancer patients from the EORTC multicentre study, analysed in Amsterdam. ▲: head and neck cancer patients from the Egyptian study, analysed in Amsterdam. □: patients from the colorectal series analysed in Umeå. The time when 50% of the tumours show divided labelled cells is a measure of the duration of G2+M. It is between 3 and 4 h for the Egyptian series and shorter than 4 h for the EORTC study difficult to determine from any published series. It is then impossible to rederive the original values. At the Gray Laboratory the average correction factor applied in a large series of patients is 0.88, but in 25% of the patients it is as low as 0.83 (GD Wilson, personal communication). At Amsterdam the average correction factor is 0.89 over a large series of patients. Table 2 would indicate such values are only applicable at labelling times beyond 6 h if $T_{G2+M}$ is 3.0 h or longer.

White and colleagues long ago recognized that any simple correction is inappropriate and have proposed a series of more complex formulae to correct with greater accuracy (White et al, 1990; Terry and Peters, 1995). Their approach is the same as the one we have adopted here and requires assumptions be made about phase length durations. They assume, however, that the duration of G2+M is 30% of S. It is clear that their approach is more accurate and will provide a truer estimate of the initial LI. Most of the practitioners of flow cytometry have found the simple practical solution more appealing than attempting to incorporate the more complex mathematics. The use of the nomogram in Table 2 or the curves in Figures 2 and 4 provide a simple means of checking if the correction factor is reasonable before it is applied.

The issue of the method of correcting LI obtained at late sampling time to the value that was relevant at time zero has recently been addressed in detail by Johansson et al (1998). Using data from in vitro experiments they have intercompared four different correction techniques to see which would give the smallest change of corrected LI with sampling time. They show marked differences between four mathematical models, three of which purport to correct for the addition by division of both unlabelled (G2) cells and labelled (S) cells. There is a 20% difference in the corrected LI that they calculate with these four formulae. Most of the corrected values differ significantly from the LI values they have actually observed with a very short labelling interval. This amply illustrates the problem but does not provide a general solution.

We have attempted in this paper to illustrate graphically the concept behind the need for a mathematical correction factor in order to demonstrate the parameters that influence the magnitude of that correction. We have illustrated that the ‘simple’ correction is unreasonable unless very short $T_{G2+M}$ values are relevant in human tumours, or quite long intervals are used between labelling and excision. Samples taken at short intervals, less than the duration of G2, are therefore most at risk from this underestimation of LI.

When large series of patients are reported, all the information about the details of time between injection and sampling is, of course, lost in the averaging procedures. Figure 6 shows that the uncorrected data are actually very close to the mathematically corrected data, since the correction factor rarely exceeds 1.1. In these studies, 29% of the tumour samples were taken at intervals

*© 1999 Cancer Research Campaign*

outside of the recommended labelling time of 4–8 h. Twenty-two per cent were taken at shorter times and 7% at longer times. The median time of biopsy was 5.3 h and the median correction factor applied was 0.73. We suggest that the provision of the raw uncorrected data should be recommended in all publications. We also suggest that every correction factor derived 'blindly' for the sample in the flow cytometer should be cross-checked against a nomogram such as those in Table 2 to see whether it is a reasonable figure, taking into account the interval between injection and surgery/biopsy. This could certainly lead to overestimations of $T_{pot}$ and a false perception of the speed of tumour cell proliferation.

### CONCLUSION

We conclude that the simple application of a gating procedure to correct LI values obtained many hours after labelling may be hazardous. In our institute it produces a 27% reduction in the median LI values compared with the raw data. This underestimation of LI in human tumours may be a common systematic artefact in other laboratories. We would urge those groups collecting LI values for the assessment of their prognostic or predictive value to reconsider the correction factors that have been applied. Application of an inappropriate correction factor, especially for the short sample times could reduce the apparent prognostic power of LI as a proliferation marker, simply because any true correlation may be obscured.

### ACKNOWLEDGEMENTS

We are grateful to Prof. Hassan Awwad for allowing us to use his flow cytometry data to derive $T_{G2}$ values. We are grateful to the Umeå Cancerforskningsfond for financial support and to Bo Littbrand and Roger Stenling for encouragement and for stimulating discussions.

### REFERENCES

Begg AC (1989) Derivation of cell kinetic parameters from human tumours after labelling with bromodeoxyuridine. *Br Inst Radiol Report* **19**: 113–119

Begg AC (1995) The clinical status of $T_{pot}$ as a predictor? Or why no tempest in the $T_{pot}$? *Int J Rad Oncol Biol Phys* **32**: 1539–1541

Begg AC, McNally NJ, Shrieve DC and Kärcher H (1985) A method to measure the duration of DNA synthesis and the potential doubling time from a single sample. *Cytometry* **6**: 620–626

Begg AC, Moonen L, Hofland I, Dessing M and Bartelink H (1988) Human tumour cell kinetics using a monoclonal antibody against iododeoxyuridine: intratumour sampling variations. *Radiother Oncol* **11**: 337–347

Begg AC, Haustermans K, Hart AAM, Dische S, Saunders M, Zackrisson B, Gustafsson H, Coucke P, Paschoud N, Hoyer M, Overgaard J, Antognoni P, Richetti A, Bourhis J, Bartelink H, Horiot JC, Corvo R, Giaretti W, Awwad H, Shouman T, Jouffroy T, Maciorowski Z, Dobrowsky W, Struikmans H and Wilson GD (1999) The value of pretreatment cell kinetic parameters as predictors for radiotherapy outcome in head and neck cancer: a multicenter analysis. *Radiother Oncol* (in press.)

Bergström C, Palmqvist R, Denekamp J, Öberg Å, Tavelin B and Stenling R (1998) Factors influencing the estimates of proliferative labelling indices in rectal cancer. *Radiother Oncol* **46**: 169–177

Bennett MH, Wilson GD, Dische S, Saunders MI, Martindale CA, Robinson BM, O'Halloran AE, Leslie MD and Laing JHE (1992) Tumour proliferation assessed by combined histological and flow cytometric analysis: implications for therapy in squamous cell carcinoma in the head and neck. *Br J Cancer* **65**: 870–878

Brons PPT, Raemaekers JMM, Bogman MJJT, Van ERP PEJ, Boezeman JBM, Pennings AHM, Wessels HMC and Haanen C (1992) Cell cycle kinetics in malignant lymphoma studied with in vivo iododeoxyuridine administration, nuclear Ki-67 staining, and flow cytometry. *Blood* **80**: 2336–2343

Dolbeare FA, Grazner HG, Pallavicini MG and Gray JW (1983) Flow cytometric measurement of total DNA content and incorporated bromodeoxyuridine. *Proc Natl Acad Sci USA* **80**: 5573–5577

Fowler JF and Lindstrom MJ (1992) Loss of local control with prolongation in radiotherapy. *Int J Rad Oncol Biol Phys* **23**: 457–467

Grazner HG (1982) Monoclonal antibody to 5-bromo- and 5-iododeoxyuridine: a new reagent for detection of DNA replication. *Science* **218**: 474–475

Howard A and Pelc SR (1953) Synthesis of deoxyribonucleic acid in normal and irradiated cells and its relation to chromosome breakage. *Heredity (Edinnburgh)* **6**: 261–273

Johansson MC, Johansson R, Baldetorp B and Oredsson SM (1998) Comparison of different labelling index formulae used on bromodeoxyuridine flow cytometry data. *Cytometry* **32**: 233–240

Palmqvist R, Öberg Å, Bergström C, Rutegård JN, Zackrisson B and Stenling R (1998) Systematic heterogeneity and prognostic significance of cell proliferation in colorectal cancer. *Br J Cancer* **77**: 917–925

Rew DA, Wilson GD, Taylor I and Weaver PC (1991) Proliferation characteristics of human colorectal carcinomas measured in vivo. *Br J Surg* **78**: 60–66

Steel GG (1977) *Growth Kinetics of Tumours*. Oxford University Press: London.

Terry NHA, Meistrich ML, Roubein LD, Lynch PM, Dubrow RA and Rich TA (1995) Cellular kinetics in rectal cancer. *Br J Cancer* **72**: 435–441

Terry NHA and Peters LJ (1995) The predictive value of tumour-cell kinetic parameters in radiotherapy. Considerations regarding data production and analysis. *J Clin Oncol* **13**: 1833–1836

White RA, Terry NHA and Meistrich ML (1990) New methods for calculating kinetic properties of cells in vitro using pulse labelling with bromodeoxyuridine. *Cell Tissue Kinet* **23**: 561–573

Wilson GD, McNally NJ, Dische S, Saunders MI, Des Rochers C, Lewis AA and Bennett MH (1988) Measurement of cell kinetics in human tumours in vivo using bromodeoxyuridine incorporation and flow cytometry. *Br J Cancer* **58**: 423–431

Wilson GD (1993) Limitations of the bromodeoxyuridine technique for measurement of tumour proliferation. In: *Medical Radiology, Current Topics in Clinical Radiobiology of Tumours*, Beck-Bornholdt HP (ed), pp. 27–43. Springer–Verlag: Berlin.

Wilson MS, West CML, Wilson GD, Roberts SA, James RD and Schofield PF (1993) An assessment of the reliability and reproducibility of measurement of potential doubling times ($T_{pot}$) in human colorectal cancers. *Br J Cancer* **67**: 754–759

Wilson MS, West CML, Wilson GD, Roberts SA, James RD and Schofield PF (1993) Intra-tumoural heterogeneity of tumour potential doubling times ($T_{pot}$) in colorectal cancer. *Br J Cancer* **68**: 501–506

© 1999 Cancer Research Campaign 

## References

[PDF_00851] Begg A. C., McNally N. J., Shrieve D. C., Kärcher H. (1985). A method to measure the duration of DNA synthesis and the potential doubling time from a single sample.. Cytometry.

[PDF_00854] Begg A. C., Moonen L., Hofland I., Dessing M., Bartelink H. (1988). Human tumour cell kinetics using a monoclonal antibody against iododeoxyuridine: intratumour sampling variations.. Radiother Oncol.

[PDF_00847] Begg A. C. (1995). The clinical status of Tpot as a predictor? Or why no tempest in the Tpot!. Int J Radiat Oncol Biol Phys.

[PDF_00867] Bennett M. H., Wilson G. D., Dische S., Saunders M. I., Martindale C. A., Robinson B. M., O'Halloran A. E., Leslie M. D., Laing J. H. (1992). Tumour proliferation assessed by combined histological and flow cytometric analysis: implications for therapy in squamous cell carcinoma in the head and neck.. Br J Cancer.

[PDF_00864] Bergström C., Palmqvist R., Denekamp J., Oberg A., Tavelin B., Stenling R. (1998). Factors influencing the estimates of proliferative labelling indices in rectal cancer.. Radiother Oncol.

[PDF_00872] Brons P. P., Raemaekers J. M., Bogman M. J., van Erp P. E., Boezeman J. B., Pennings A. H., Wessels H. M., Haanen C. (1992). Cell cycle kinetics in malignant lymphoma studied with in vivo iododeoxyuridine administration, nuclear Ki-67 staining, and flow cytometry.. Blood.

[PDF_00876] Dolbeare F., Gratzner H., Pallavicini M. G., Gray J. W. (1983). Flow cytometric measurement of total DNA content and incorporated bromodeoxyuridine.. Proc Natl Acad Sci U S A.

[PDF_00879] Fowler J. F., Lindstrom M. J. (1992). Loss of local control with prolongation in radiotherapy.. Int J Radiat Oncol Biol Phys.

[PDF_00881] Gratzner H. G. (1982). Monoclonal antibody to 5-bromo- and 5-iododeoxyuridine: A new reagent for detection of DNA replication.. Science.

[PDF_00886] Johansson M. C., Johansson R., Baldetorp B., Oredsson S. M. (1998). Comparison of different labelling index formulae used on bromodeoxyuridine-flow cytometry data.. Cytometry.

[PDF_00889] Palmqvist R., Oberg A., Bergström C., Rutegård J. N., Zackrisson B., Stenling R. (1998). Systematic heterogeneity and prognostic significance of cell proliferation in colorectal cancer.. Br J Cancer.

[PDF_00892] Rew D. A., Wilson G. D., Taylor I., Weaver P. C. (1991). Proliferation characteristics of human colorectal carcinomas measured in vivo.. Br J Surg.

[PDF_00895] Terry N. H., Meistrich M. L., Roubein L. D., Lynch P. M., Dubrow R. A., Rich T. A. (1995). Cellular kinetics in rectal cancer.. Br J Cancer.

[PDF_00897] Terry N. H., Peters L. J. (1995). The predictive value of tumor-cell kinetic parameters in radiotherapy: considerations regarding data production and analysis.. J Clin Oncol.

[PDF_00900] White R. A., Terry N. H., Meistrich M. L. (1990). New methods for calculating kinetic properties of cells in vitro using pulse labelling with bromodeoxyuridine.. Cell Tissue Kinet.

[PDF_00903] Wilson G. D., McNally N. J., Dische S., Saunders M. I., Des Rochers C., Lewis A. A., Bennett M. H. (1988). Measurement of cell kinetics in human tumours in vivo using bromodeoxyuridine incorporation and flow cytometry.. Br J Cancer.

[PDF_00911] Wilson M. S., West C. M., Wilson G. D., Roberts S. A., James R. D., Schofield P. F. (1993). An assessment of the reliability and reproducibility of measurement of potential doubling times (Tpot) in human colorectal cancers.. Br J Cancer.

[PDF_00916] Wilson M. S., West C. M., Wilson G. D., Roberts S. A., James R. D., Schofield P. F. (1993). Intra-tumoral heterogeneity of tumour potential doubling times (Tpot) in colorectal cancer.. Br J Cancer.

